# Co-Expression of GRK2 Reveals a Novel Conformational State of the µ-Opioid Receptor

**DOI:** 10.1371/journal.pone.0083691

**Published:** 2013-12-20

**Authors:** Sarah A. Nickolls, Sian Humphreys, Mellissa Clark, Gordon McMurray

**Affiliations:** Neusentis, A Pfizer Research Unit, Granta Park, Cambridge, United Kingdom; Medical School of Hannover, Germany

## Abstract

Agonists at the µ-opioid receptor are known to produce potent analgesic responses in the clinical setting, therefore, an increased understanding of the molecular interactions of ligands at this receptor could lead to improved analgesics. As historically morphine has been shown to be a poor recruiter of β-arrestin in recombinant cell systems and this can be overcome by the co-expression of GRK2, we investigated the effects of GRK2 co-expression, in a recombinant µ-opioid receptor cell line, on ligand affinity and intrinsic activity in both β-arrestin recruitment and [^35^S]GTPγS binding assays. We also investigated the effect of receptor depletion in the β-arrestin assay. GRK2 co-expression increased both agonist Emax and potency in the β-arrestin assay. The increase in agonist potency could not be reversed using receptor depletion, supporting that the effects were due to a novel receptor conformation not system amplification. We also observed a small but significant effect on agonist K_L_ values. Potency values in the [^35^S]GTPγS assay were unchanged; however, inverse agonist activity became evident with GRK2 co-expression. We conclude that this is direct evidence that the µ-opioid receptor is an allosteric protein and the co-expression of signalling molecules elicits changes in its conformation and thus ligand affinity. This has implications when describing how ligands interact with the receptor and how efficacy is determined.

## Introduction

Morphine is a potent analgesic of great clinical utility. It exerts both its analgesic effects and its dose limiting adverse events through the µ-opioid receptor, a G protein coupled receptor (GPCR). Like other GPCRs, the µ-opioid receptor can couple to more than one signalling pathway and exhibits a phenomena known as biased agonism, where different agonists differ in their ability to activate different signalling pathways. The concept of biased agonism at GPCRs has been emerging over the last decade and a half. Early studies by Berg et al [1] showed changes in the rank order of efficacy depending on which G protein mediated response was measured at the 5-HT_2A_ and 5-HT_2C_ receptors. Agonist efficacies were also shown to differ in rank order at the β-adrenergic receptor when fused to different Gα subunits [2] and at the D_2_ receptor when co-expressed with different G proteins in Sf9 cells [3].

As well as activating G proteins, GPCRs are phosphorylated by G protein receptor kinases (GRKs) and this leads to the recruitment of β-arrestin and receptor internalisation [4]. However, as data emerged showing that β-arrestin is a signalling protein in its own right [5], divergent agonist profiles have also been discovered for additional signalling pathways. For instance, ligands that had been previously characterised as inverse agonists at the β2-adrenergic receptor were shown to be partial agonists for the activation of MAPK [6] and an angiotensin II analogue which does not cause G protein recruitment to the Ang_1A_ receptor, does cause β-arrestin dependent ERK1/2 activation [7]. Therefore, due to the complexity of the signalling pathways activated and the propensity of ligands to signal in a biased fashion [8], the definition of ligand – receptor interactions must be viewed as multifaceted. As such, increasing our understanding of how compounds interact with the µ-opioid receptor may lead to the discovery of analgesics with improved efficacy and/or decreased on-target adverse events.

In humans there are seven different GRKs divided into three different families (reviewed in [9]). GRK2, which together with GRK3, forms the GRK2-like family is ubiquitously expressed and has been shown to phosphorylate the µ-opioid receptor [9], [10]. This effect of GRK2 may have great importance in the in vivo translation of a ligands ability to recruit β-arrestin, as in vitro, morphine has been shown to be a weak recruiter of β-arrestin [12]–[15]. However, morphines ability to recruit β-arrestin in recombinant systems can be increased by co-expressing GRK2 [15], [16]. β-arrestin knockout mice show decreased constipation and respiratory depression, in response to morphine [17], [18], so it may be considered advantageous to boost the ability of µ-opioid agonists to recruit β-arrestin when studying their actions in recombinant systems. This is particularly true, as the recruitment and subsequent activation of β-arrestin by µ-opioid receptor agonists may underlie some of the undesirable clinical side effects seen with opioids. This is one of the avenues currently being investigated by the drug discovery industry and Trevena have recently disclosed the compound TRV130, which is currently in phase 1b clinical trials and reported to be G protein biased [19].

In this study we have investigated the effect of GRK2 co-expression on the ability of agonists to recruit β-arrestin in a U2OS µ-opioid receptor cell background. This is a commercially available cell line (DiscoveRx), in which the receptor is tagged with small enzyme fragment ProLink™ and co-expressed in cells expressing a fusion protein of β-arrestin2 and a large, N-terminal deletion mutant of β-galactosidase. When the receptor and β-arrestin interact a complete enzyme is formed, which can be detected by the addition of a chemilumiscent reagent. We choose this cell line as it is a high-throughput way of measuring β-arrestin recruitment to the µ-opioid receptor, whilst also allowing classical G protein signalling to be measured in the form of a [^35^S]GTPγS assay. When we co-expressed GRK2, as expected β-arrestin recruitment was enhanced; in addition, as we show herein, it also revealed a distinct affinity state of the receptor, providing strong evidence that signalling molecules change receptor conformation and these changes are reflected in changes in agonist affinity.

## Methods

### Cell culture

µ-opioid β-arrestin U2OS cells were purchased from DiscoveRx (Carlsbad, CA). Cells were grown in MEM modified Eagles Medium, containing 2 mM glutamax, 10% FBS, 500 µg/mL G418 and 250 µg/mL hygromycin at 37°C and 5% CO_2_ in a humidified incubator. Cells were plated in T225 flasks at a density of 3×10^6^ cells/flask, and split as required. For experiments ascertaining the effect of pertussis toxin cells were incubated for 24 h with 100 ng/ml PTX, a condition which was able to completely block any agonist mediated [^35^S]GTPγS binding after membranes were prepared.

### Transduction

A modified baculovirus system was used to express GFP or GRK2 under a CMV promoter in mammalian cells (Bacmam® Life technologies, Grand Island, NY). Cells were resuspended at a density of 3.75×10^5^ cells/ml and either GRK2 Bacman® (2.9×10^8^ pfu/ml) or GFP Bacmam® (2×10^8^ pfu/ml), at a multiplicity of infection (moi) of 5 was added to the cells, before plating as required for assay or membrane preparation, both of which were performed 24 h post-transduction. At the time of membrane preparation a morphine concentration response curve was performed in the DiscoveRx β-arrestin assay to confirm that the cells had been efficiently transduced.

### β-arrestin assay

Cells were plated at 7000 cells/well in white 384 well TC plates and incubated overnight. Cells were then treated with 1 or 10 nM β-funaltrexamine in growth media, or growth media alone, for 30 minutes at 37°C. Cells were then washed 3X with growth media (5 minutes per wash) before treatment with compounds. The agonist-loaded cell plates were then incubated at 37°C for 90 minutes, before detection of the β-arrestin receptor interaction using the DiscoveRx PathHunter detection kit (DiscoveRx, Birmingham, UK) according to the manufacturer's instructions.

### Membrane preparation

Cells were grown in T225 flasks up to 80% confluency in full growth medium. Cells were transduced 24 h before membrane preparation as required. The cell layer was washed 3X with PBS and cells were detached from the flasks using enzyme-free cell dissociation buffer, resuspended in full growth medium and centrifuged for 5 minutes at 1,000 g before being washed once with PBS. Cells were resuspended in ice-cold buffer (20 mM HEPES, 1 mM MgCl_2_). Cells were homogenized at 4°C using an T25 basic IKA® ultraturax (6×5 sec blasts on the maximal setting). The homogenate was centrifuged for 20 minutes at 1, 000 g, the supernatant was collected and then centrifuged at 55, 000 g (4°C) for 45 minutes. The resulting pellet was resuspended in buffer, and aliquots were stored at −80°C. Protein concentration was determined using the Bradford assay (Sigma-Aldrich, Gillingham, UK), using BSA as standard.

### [^3^H]-diprenorphine binding

Cell membranes (5 µg of protein) were incubated in duplicate with 1 nM [^3^H]-diprenorphine for competition binding and between 0.01 and 10 nM [^3^H]-diprenorphine for saturation binding, in a total volume of 200 µL of buffer (50 mM Tris-Cl pH 7.4, 3 mM MgCl_2_, 0.2 mM EDTA, 100 mM NaCl, 100 µM GTP, 0.5% BSA). Non-specific binding was determined by the inclusion of 1 µM naloxone. The reaction was initiated by the addition of membranes, and the plates were incubated at 25°C for 2 hours. The reaction was terminated by rapid filtration using a vacuum harvester with two 2 mL washes of ice-cold wash buffer (50 mM Tris-Cl pH 7.4, 3 mM MgCl_2_, 0.2 mM EDTA, 100 mM NaCl, 0.5% BSA). The filters were soaked in 50 µL of scintillation fluid, and the amount of radioactivity present was determined by liquid scintillation counting.

### [^3^H]-DAMGO binding

Cell membranes (5 µg of protein) were incubated in duplicate with 1 nM [^3^H]-DAMGO for competition binding and between 0.01 and 10 nM [^3^H]-DAMGO for saturation binding, in a total volume of 200 µL of buffer (50 mM Tris-Cl pH 7.4, 3 mM MgCl_2_, 0.2 mM EDTA, 100 mM NaCl, 0.5% BSA). Non-specific binding was determined by the inclusion of 1 µM naloxone. The reaction was initiated by the addition of membranes, and the plates were incubated at 25°C for 2 hours. The reaction was terminated by rapid filtration using a vacuum harvester with two 2 mL washes of ice-cold wash buffer (50 mM Tris-Cl pH 7.4, 3 mM MgCl_2_, 0.2 mM EDTA, 100 mM NaCl, 0.5% BSA). Reactions were initiated, incubated, terminated and read as above.

### [^35^S]GTPγS binding

Cell membranes (5 µg of protein) were incubated in duplicate with 0.1 nM [^35^S]GTPγS in a total volume of 200 µL of buffer (50 mM Tris-Cl pH 7.4, 3 mM MgCl_2_, 0.2 mM EDTA, 100 mM NaCl, 1 µM GDP, 0.5% BSA) in WGA Flashplates (PerkinElmer, Cambridge, UK). The reaction was initiated by the addition of membranes, and the plates were incubated at 30°C for 90 minutes. The reaction was terminated by centrifugation and membrane-bound radioactivity was determined by scintillation counting.

### Quantitative PCR

Twenty-four hours after GRK2 induction, RNA was extracted from cell pellets (approximately 2.6×10^6^ cells) using the RNeasy mini kit (Qiagen, Hilden, Germany). Human frontal lobe and cerebellum control mRNA was obtained from AMS Biotechnology (Abingdon, UK). cDNA was synthesised using the Omniscript reverse transcription kit (Qiagen, Hilden, Germany). Relative GRK expression was determined on 250 ng cDNA using quantitative PCR reagents (Life Technologies, Paisley, UK), normalised to beta actin expression levels.

### Data analysis

Data were analyzed using PRISM (GraphPad Software Inc., San Diego, CA). K_i_ values were calculated from IC_50_ values using the method of Cheng and Prusoff [20].

### Drugs and Chemicals used

[^3^H]-diprenorphine, [^3^H]-DAMGO and [^35^S]GTPγS were obtained from PerkinElmer Life Sciences (Cambridge, UK), β-funaltrexamine, oxycodone, [D-Ala^2^, MePhe^4^, gly^5^ –ol]-enkephalin (DAMGO), endomorphin 1 and 2, morphine and pertussis toxin were obtained from Sigma-Aldrich. Pfizer standard 1 (compound 3b, 2-(L-Tyrosylamino)-1-[(*N*-acetyl-L-phenylalanyl)-amino]-2-methylpropane hydrochloride [21] was synthesised in-house.

## Results

The aim of this study was to evaluate the effect of GRK2 co-expression on the affinity and intrinsic activity of µ-opioid ligands. We used Bacman® transduction to express GRK2 in a DiscoveRx U2OS- µ-opioid receptor β-arrestin stable cell line, and performed concentration effect curves using the DiscoveRx β-arrestin assay for a variety of agonists, which had previously been shown to give responses ranging from partial to full agonism in untransduced U2OS- µ-opioid receptor β-arrestin cells.

### GRK2 expression effects agonist parameters in the β-arrestin assay

**Figure 1:**
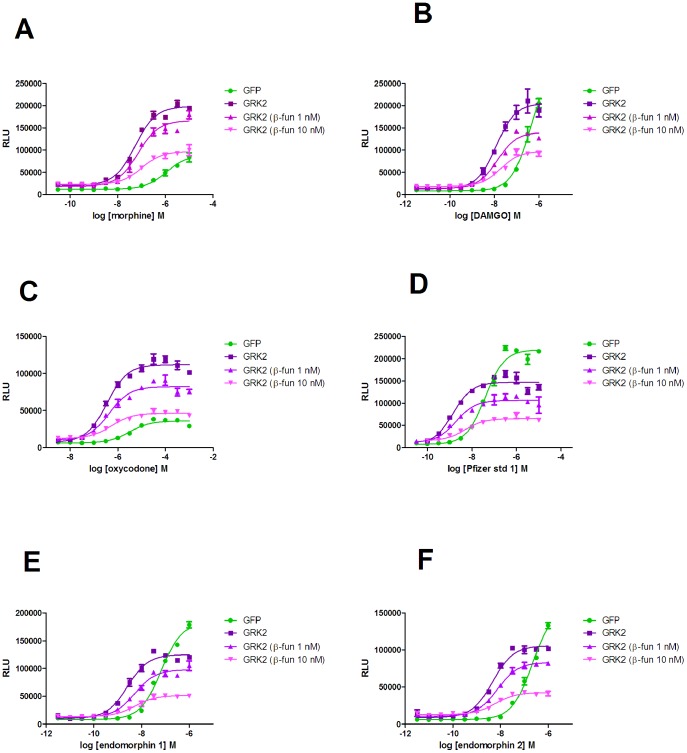
The effect of co-expression of GRK2 and subsequent β-funaltrexamine treatment on β-arrestin recruitment in U2OS µ-opioid receptor β-arrestin cells. Concentration-effect curves for DAMGO, morphine, Pfizer standard 1, endomorphin-1, endomorphin-2 and oxycodone were performed in U2OS µ-opioid receptor β-arrestin cells which had been transduced with either GFP or GRK2. Cells transduced with GRK2 were also treated with either 1 nM or 10 nM β-funaltrexamine before concentration-effect curves were determined. Experiments were performed as described in the experimental section and data are representative of four independent experiments.

As a control for Bacmam® transduction, cells were also transduced with GFP. The co-expression of GFP was ∼100% as observed with confocal microscopy and had no effect on the potency or intrinsic activity of compounds, but did decrease the maximal relative luminescence of the assay (by about 20%), which was considered to a slight cytotoxic effect. In line with this when a higher moi was used the maximal assay luminescence was further decreased, again with no change in agonist parameters (data not shown). However, the decrease in the maximal luminescence observed with GFP (moi of 5) was not as great as that observed with GRK2 (moi of 5), which was approximately 40%. This made agonists with a high Emax in the presence of GFP appear to reach a lower Emax in the presence of GRK2, therefore Emax data was normalised to that of DAMGO in each respective condition to allow accurate comparison (Table 1). GRK2 co-expression also slightly increased basal luminescence (Figure 1), indicative of an increased interaction between receptor and β-arrestin in the presence of GRK2. As predicted, GRK2 increased the Emax of morphine in the β-arrestin recruitment assay. At lower mois a partial increase was observed, but increasing the moi above 5 did not result in any further enhancement of intrinsic activity or potency. This led us to believe that all ∼100% cells were transduced using an moi of 5, although increasing the moi did further reduce the maximal luminescence further, in line with the data obtained by transducing with Bacman® expressing GFP (data not shown).

**Table 1:** The effect of co-expression of GRK2 and subsequent treatment with β-funaltrexamine on the potency and intrinsic activity of µ-opioid receptor agonists in a β-arrestin recruitment assay.

	GFP	GFP	GRK2	GRK2	GRK2	GRK2	GRK2	GRK2
	Untreated pEC_50_±s.e.m (EC_50_ nM)	Emax	Untreated pEC_50_±s.e.m (EC_50_ nM)	Emax	β-fun 1 nM pEC_50_±s.e.m (EC_50_ nM)	% decrease in Emax comp to untreated	β-fun 10 nM pEC_50_±s.e.m (EC_50_ nM)	% decrease in Emax comp to untreated
DAMGO	6.16±0.14 (692)	100%	7.64±0.15^A^ (22.9)	100%	7.50±0.16 (31.6)	64±12	7.48±0.12 (33.1)	36±4
Pfizer comp 1	7.59±0.17 (25.7)	106±4	9.04±0.10^A^ (0.912)	99±8	8.86±0.09 (1.38)	71±9	8.65±0.16 (2.24)	46±6
Morphine	6.08±0.09 (832)	26±2	7.29±0.07^A^ (51.2)	98±5	7.12±0.02 (75.6)	65±8	6.95±0.03 (112)	42±6
Oxycodone	5.25±0.22 (5620)	20±3	6.28±0.15^A^ (525)	95±4	6.18±0.12 (661)	61±8	6.06±0.12 (871)	39±3
Endomorphin 1	6.87±0.18 (135)	78±7	8.45±0.07^A^ (3.55)	76±9	8.16±0.09 (6.17)	67±7	8.10±0.07 (7.94)	36±1
Endomorphin 2	6.56±0.11 (275)	69±3	8.21±0.07^A^ (6.17)	74±4	8.05±0.12 (8.91)	59±9	8.07±0.07 (8.51)	31±3

Functional properties of agonists in the β-arrestin assay were determined as described in the experimental section. Potency values before and after treatment with β-funaltrexamine are expressed as pEC_50_±s.e.m (EC_50_ nM). Emax values before β-funaltrexamine treatment are expressed as % of the maximal response of DAMGO in U2OS µ-opioid receptor β-arrestin cells co-expressing either GFP or GRK2 (mean±s.e.m). The percentage reduction in Emax after treatment is expressed as a percentage of the agonist's Emax before treatment (mean±s.e.m). Data are from four independent experiments.

A Values are significantly different from those determined in the absence of GRK2 (p<0.05, two-tailed t-test).

GRK2 expression, however, also significantly increased the potency of all of the agonists tested (p<0.05 two-tailed unpaired t-test). Furthermore, its effect on the Emax of compounds was not consistent. The weak partial agonists (oxycodone and morphine), appeared as full agonists in the presence of GRK2, but the stronger partial agonists (endomorphin 1 and endomorphin 2) did not show a GRK2 dependent change in Emax. The antagonist naloxone had no effect on β-arrestin recruitment in either the absence or presence of GRK2 co-expression.

### The change in agonist properties in the β-arrestin assay is not due to system amplification

Some of these data might infer that amplification had been introduced into the system, in line with the operational model of agonism [22], in which downstream amplification or increasing receptor density leads to enhanced agonist potency and intrinsic activity. However, two pieces of data are inconsistent with this explanation. Firstly, there was no increase in the intrinsic activity of the endomorphins in the presence of GRK2. Secondly we have previously shown that there is a linear relationship between occupancy and response in the DiscoveRx β-arrestin assay, which is independent of receptor density [23]. Therefore, β-funaltrexamine (an irreversible antagonist of the µ-opioid receptor [24]) was used to deplete the number of receptors available for agonist activation in GRK2 expressing U2OS µ-opioid β-arrestin cells. If GRK2 expression has caused system amplification, this would be predicted to decrease the potency and intrinsic activity of agonists in the β-arrestin assay. Treatment of cells with either 1 or 10 nM β-funaltrexamine led to a significant decrease in agonist Emax, (Figure 1), but very little change in EC_50_ (Table 1). The initial intrinsic activity and potency of the compound had no effect on the resultant Emax after treatment. These data are consistent with the linear relationship between the receptor and β-arrestin in the DiscoveRx assay and are therefore indicative that GRK2 co-expression has changed the receptor conformation measured in the β-arrestin assay, rather than caused amplification.

### GRK2 increases the affinity of agonists, but not antagonists, for the low affinity state

**Figure 2:**
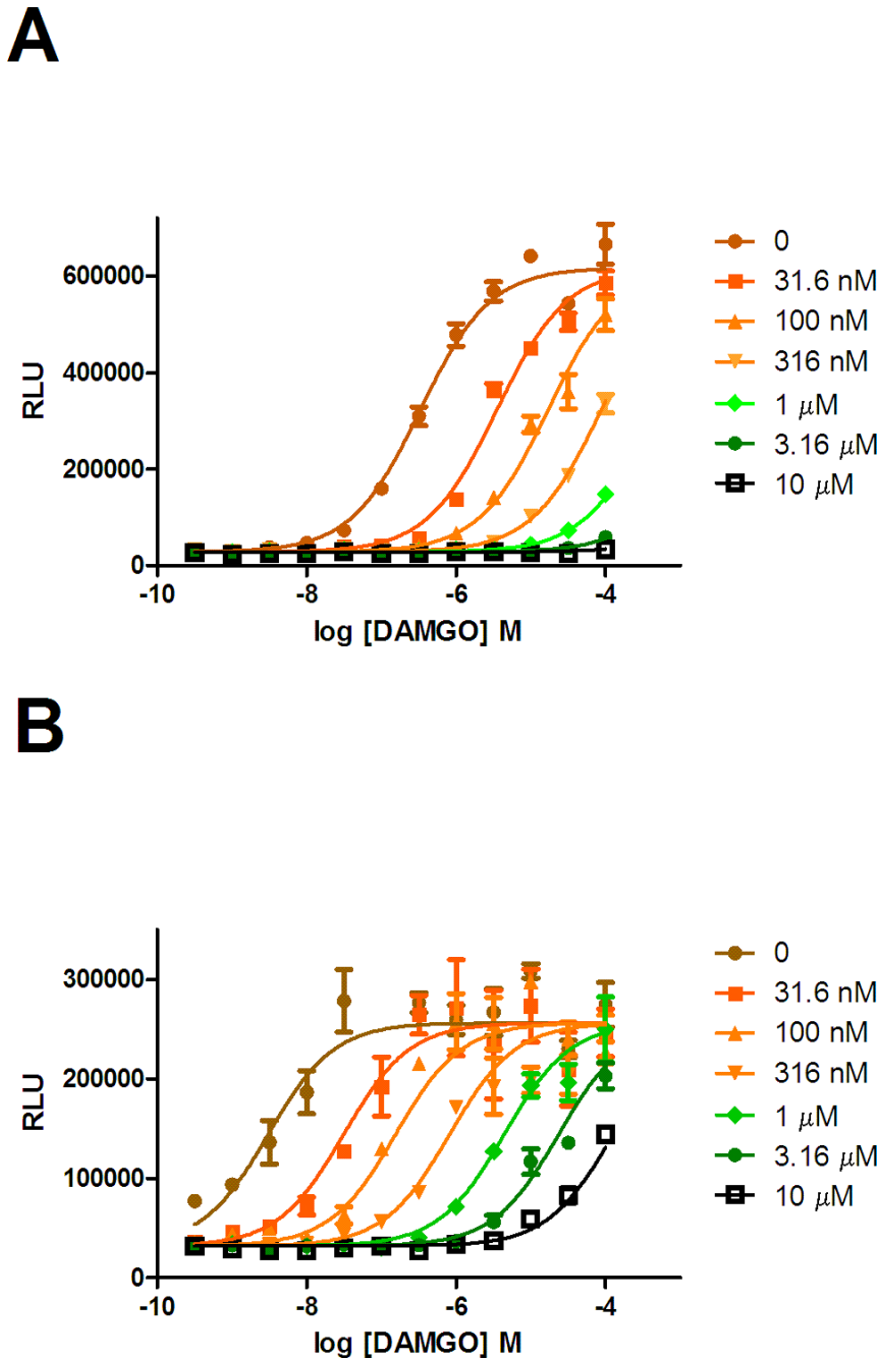
The effect of naloxone on the ability of DAMGO to recruit β-arrestin. Concentration effect curves for DAMGO were determined in the absence or presence of 31.6 µM, 3.16 µM or 10 µM naloxone in U2OS µ-opioid receptor β-arrestin cells in the absence (A) or presence (B) of co-expressed GRK2.

**Figure 3:**
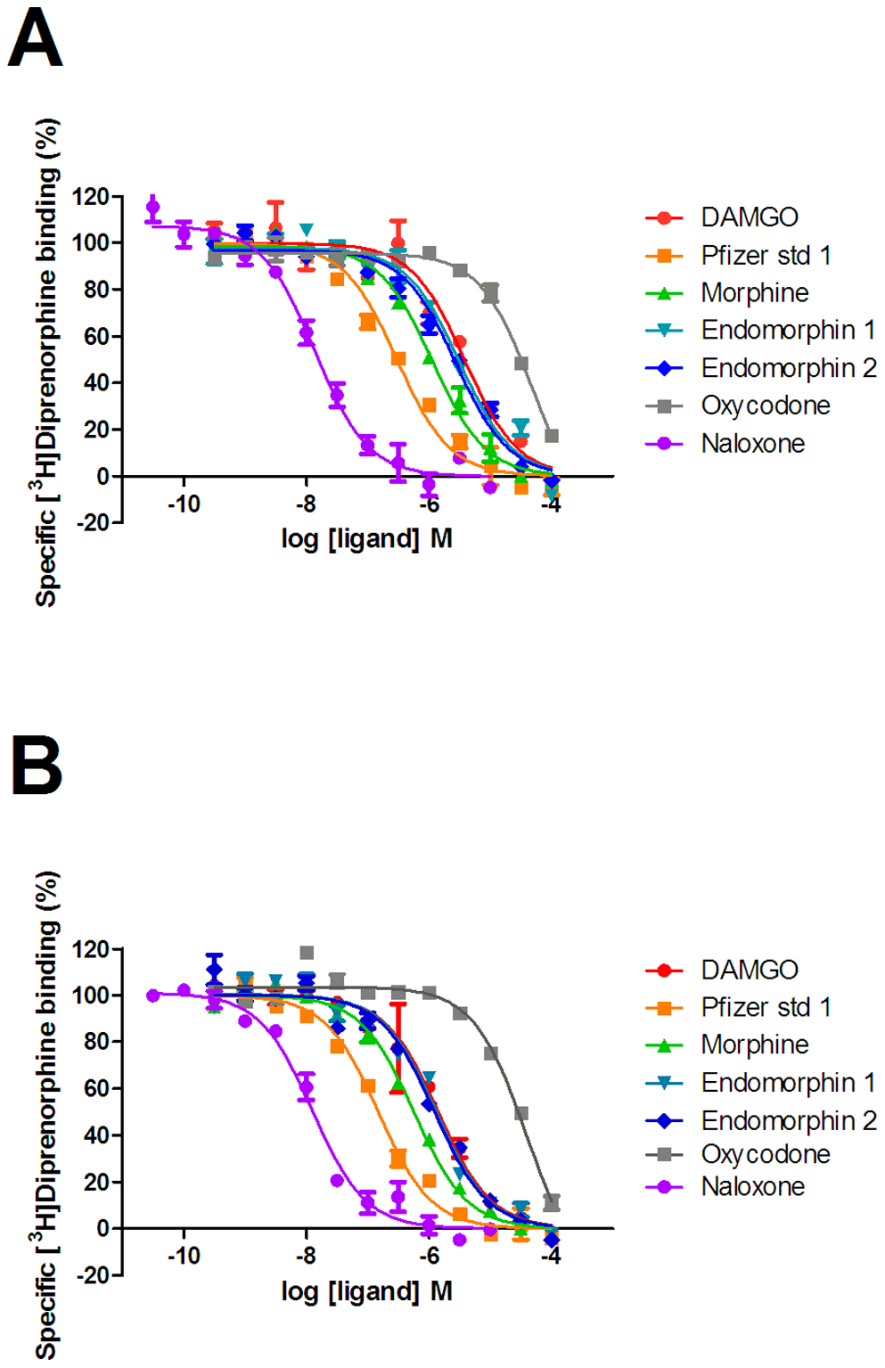
Competition binding of ligands to the low affinity state of the µ-opioid receptor. Competition binding experiments versus [^3^H]diprenorphine were performed as described in the experimental section to membranes prepared from U2OS µ-opioid receptor β-arrestin cells in the absence (A) or presence (B) of co-expressed GRK2. Graphs are representative of three independent experiments.

**Figure 4:**
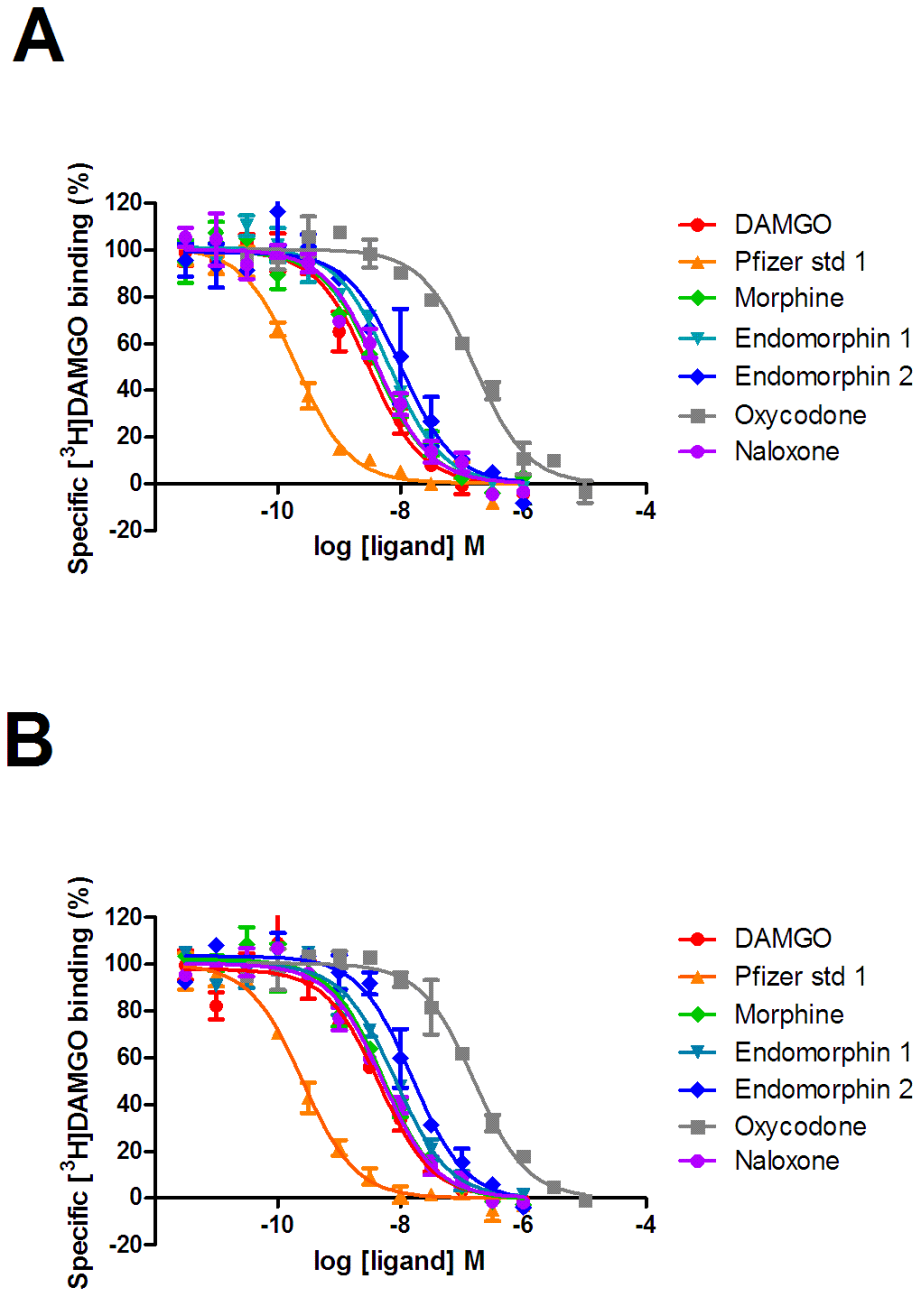
Competition binding of ligands to the high affinity state of the µ-opioid receptor. Competition binding experiments versus [^3^H]DAMGO were performed as described in the experimental section to membranes prepared from U2OS µ-opioid receptor β-arrestin cells in the absence (A) or presence (B) of co-expressed GRK2. Graphs are representative of four independent experiments.

Subsequently, we investigated the ability of naloxone to inhibit µ-opioid receptor mediated β-arrestin recruitment in the absence or presence of GRK2 co-expression. Naloxone was able to inhibit β-arrestin recruitment in a competitive manner (Figure 2) and there was no change in pA_2_ in either condition (absence of GRK2 coexpression 8.22±0.17; presence of GRK2 co-expression 8.23±0.09, mean±s.e.m n = 4). These results were consistent with a selective increase in the affinity of agonists, but not antagonists, when GRK2 was co-expressed. Therefore, we investigated the binding of compounds to membranes prepared from cells in the absence or presence of co-expressed GRK2. Both low affinity binding (K_L_) using the antagonist radioligand [^3^H]diprenorphine in the presence of guanine nucleotides (Figure 3) and high affinity binding (K_H_) using the agonist radioligand [^3^H]DAMGO (Figure 4) were studied. Saturation binding revealed that there was no change in Kd for either radioligand ([^3^H]-DAMGO control 0.69±0.32 nM, GRK2 0.99±0.56 nM; [^3^H]-diprenorphine control 0.15±0.03 nM, GRK2 0.17±0.01 nM n = 3) and that the expression of the µ-opioid receptor was broadly equivalent in both preparations (Bmax [^3^H]-diprenorphine control 4.17±1.22 pmol/mg, GRK2 4.94±1.04 pmol/mg; [^3^H]-DAMGO control 1.61±0.53 pmol/mg; GRK2 2.39±0.64 pmol/mg, n = 3). We observed no significant difference in the K_H_ value of any ligand when GRK2 was co-expressed (Table 2). However, there was a small but significant increase in the K_L_ of most agonists (p<0.05 two-tailed unpaired t-test), with the exception of morphine. There was no difference in the affinity of the antagonist naloxone when GRK2 was co-expressed.

**Table 2:** The effect of GRK2 co-expression on the affinity of µ-opioid receptor ligands.

	Untransduced pK_L_±s.e.m (K_L_ nM)	GRK2 moi 5 pK_L_±s.e.m (K_L_ nM)	Untransduced pK_H_ ±s.e.m (K_H_ nM)	GRK2 moi 5 K_H_±s.e.m (K_H_ nM)
DAMGO	6.23±0.10 (589)	6.65±0.04^A^ (224)	9.16±0.10 (0.692)	9.16±0.17 (0.692)
Pfizer comp 1	7.37±0.08 (42.7)	7.66±0.06^A^ (21.9)	10.32±0.13 (0.0479)	10.29±0.17 (0.0513)
Morphine	6.69±0.20 (204)	6.96±0.03 (110)	9.05±0.10 (0.891)	8.85±0.28 (1.41)
Oxycodone	5.12±0.05 (7590)	5.25±0.07^A^ (5623)	7.48±0.18 (33.1)	7.54±0.20 (28.8)
Endomorphin 1	6.31±0.08 (490)	6.65±0.06^A^ (224)	8.90±0.15 (1.26)	8.91±0.22 (1.23)
Endomorphin 2	6.40±0.08 (398)	6.71±0.07^A^ (195)	8.66±0.15 (2.19)	8.58±0.20 (2.63)
Naloxone	8.65±0.04 (2.24)	8.64±0.06 (2.29)	9.24±0.18 (0.575)	9.09±0.12 (0.813)

Affinity for the low affinity site (K_L_) values were determined in competition binding assays versus [^3^H]diprenorphine in the presence of sodium ions and GTP, as described in the experimental section. Values are mean±s.e.m of three independent experiments. Affinity for the high affinity site (K_H_) values were determined in competition binding assays versus [^3^H]DAMGO in the presence of sodium ions, as described in the experimental section. Values are mean±s.e.m of four independent experiments.

A Values are significantly different from those determined in the absence of GRK2 (p<0.05, two-tailed t-test).

### Naloxone is an inverse agonist in a [^35^S]GTPγS binding assay when GRK2 is co-expressed

**Figure 5:**
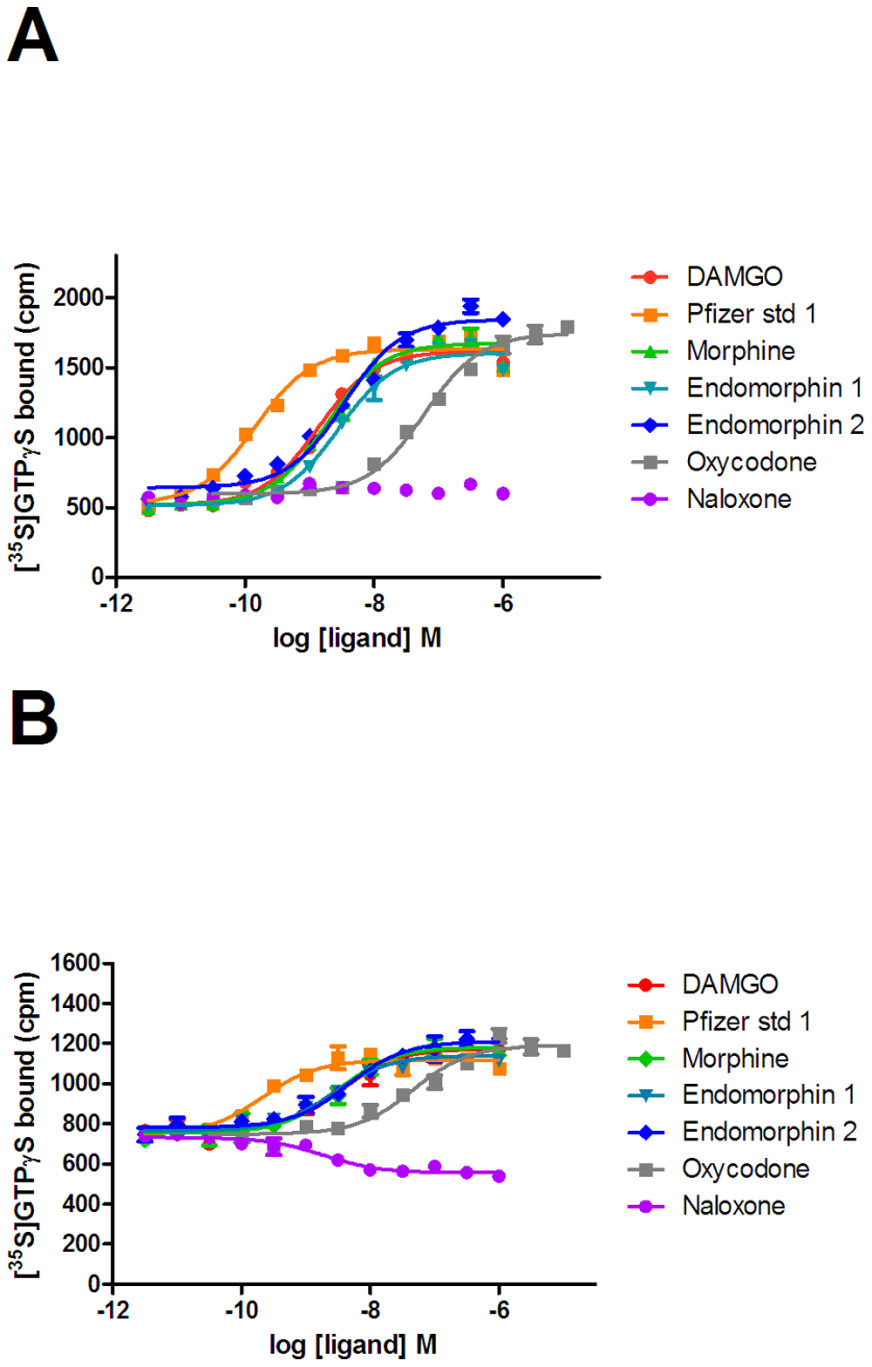
The ability of ligands to activate G proteins through the µ-opioid receptor. Agonist stimulated [^35^S]GTPγS binding to membranes prepared from U2OS µ-opioid receptor β-arrestin cells in the absence (A) and presence (B) of co-expressed GRK2 was measured. Concentration-effect curves ligands were determined as described in the experimental system. Graphs are representative of four independent experiments.

We then examined the ability of µ-opioid receptor ligands to stimulate [^35^S]GTPγS binding in membranes prepared from cells in the absence or presence of co-expressed GRK2 (Figure 5). Basal [^35^S]GTPγS binding was increased in membranes prepared from cells transduced with GRK2 compared to untransduced cells (577±94 cpm compared with 488±53 cpm, n = 4) and the overall assay window was decreased (max binding 1002±105 cpm compared with 1422±165 cpm, n = 4). However, all agonists were able to stimulate [^35^S]GTPγS binding in a concentration dependent manner in both membrane preparations and all agonists tested appeared as full agonists in both preparations (Table 3). There was no significant change in agonist potencies between the two preparations, although there was a slight trend for a lower value in the presence of GRK2. Conversely, naloxone was a neutral antagonist in membranes from cells prepared from untransduced cells, but was clearly an inverse agonist in membranes from cells which had been transduced with GRK2.

**Table 3:** The effect of GRK2 co-expression on the potency and intrinsic activity of µ-opioid receptor ligands in a [^35^S]GTPγS assay.

	untransduced	untransduced	GRK 2	GRK 2
	pEC_50_±s.e.m (EC_50_ nM)	Emax	pEC_50_±s.e.m (EC_50_ nM)	Emax
DAMGO	8.75±0.10 (1.78)	100	8.77±0.11 (1.70)	100
Pfizer comp 1	9.66±0.11 (0.219)	103±4	9.77±0.11 (0.170)	100±4
Morphine	8.54±0.10 (2.88)	103±3	8.62±0.06 (2.40)	107±3
Oxycodone	7.17±0.06 (67.6)	95±6	7.36±0.24 (43.7)	104±2
Endomorphin 1	8.31±0.18 (4.90)	97±3	8.53±0.11 (2.95)	93±1
Endomorphin 2	8.43±0.17 (3.72)	104±4	8.64±0.16 (2.29)	101±2
Naloxone	ND	NQ	8.56±0.07 (2.75)	−15±3

Functional properties of µ-opioid receptor ligands in the [^35^S]GTPγS binding assay were determined as described in the experimental section. Potency values are expressed as pEC_50_±s.e.m (EC_50_ nM). Emax values are expressed as % of the maximal response of DAMGO (mean±s.e.m). Data are from four independent experiments.

### Pertussis toxin only effects β-arrestin recruitment in the absence of GRK2 co-expression

**Figure 6:**
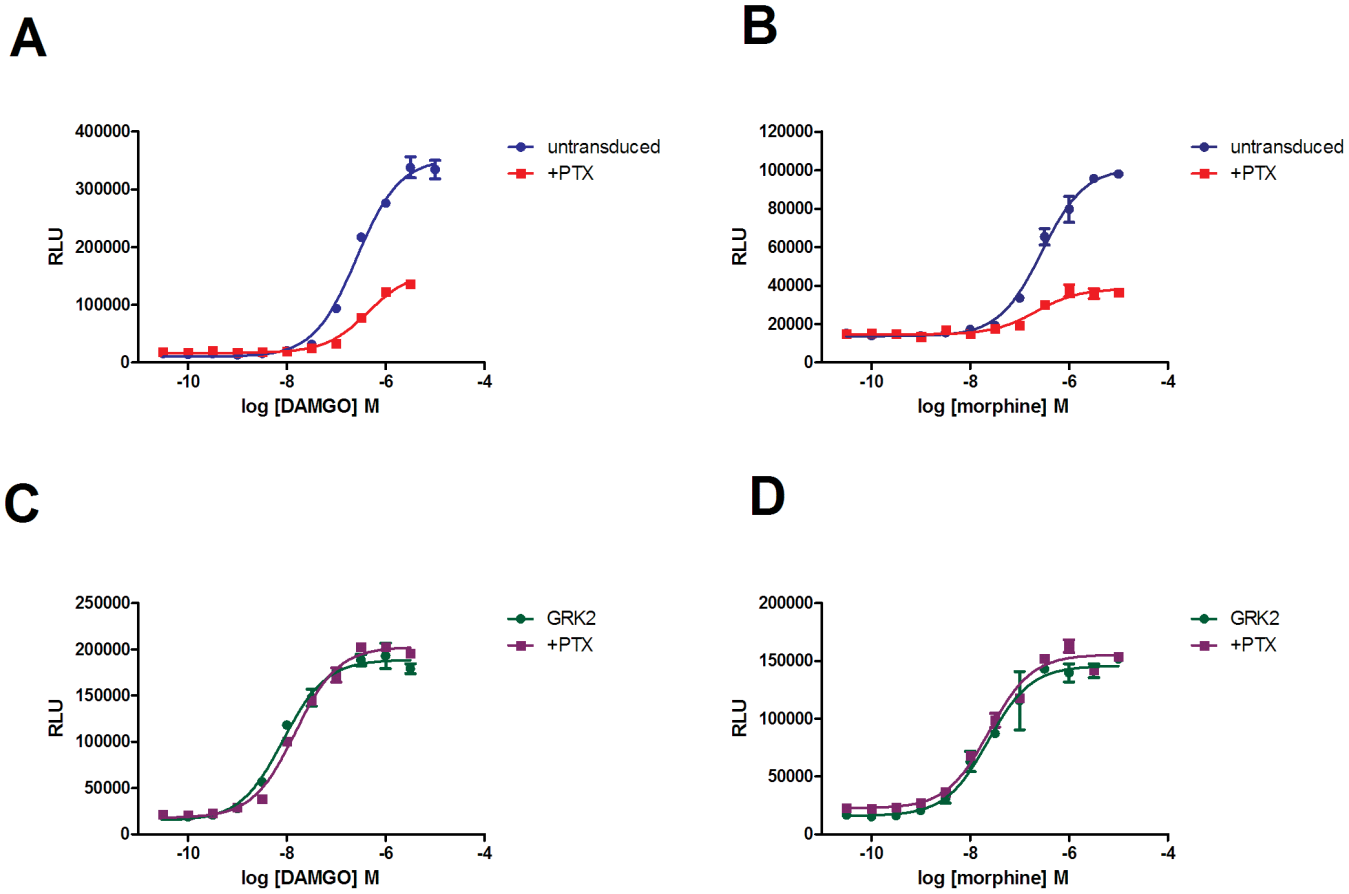
The effect of PTX on the ability of DAMGO and morphine to recruit β-arrestin in U2OS µ-opioid receptor β-arrestin cells. Concentration response curves were performed in the absence (▪) or presence (•) of PTX, in untransduced cells (graphs a and b) or cells co-expressing GRK2 (graphs c and d).

Furthermore, we investigated the effect of pertussis toxin on the intrinsic activity and potency of µ-opioid receptor agonists in the β-arrestin assay in the absence or presence of GRK2 co-expression. In untransduced cells approximately 60–70% of β-arrestin recruitment was PTX sensitive whichever agonist (DAMGO, Pfizer standard-1, morphine, endomorphin-1/-2, oxycodone) was investigated (Figure 6 – morphine and DAMGO curves shown as representative). Conversely, in cells co-expressing GRK2, PTX treatment had no observable effect on the recruitment of β-arrestin.

### There is little GRK expression in the absence of transduction in U2OS cells

**Figure 7:**
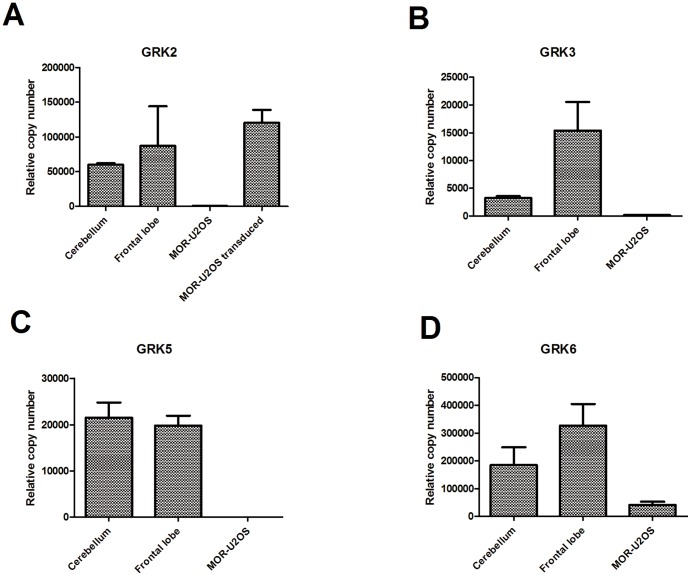
Taqman analysis of endogenous GRK expression in U2OS µ-opioid receptor β-arrestin cells. Data were generated as described in the experimental section and are plotted as mean±s.e.m.

Finally, we investigated which GRKs were endogenously expressed in U2OS µ-opioid receptor cells by quantitative PCR (Figure 7). mRNA for all the GRKs was detectable, but there was very low levels of GRK mRNA relative to expression in either the cerebellum or frontal lobe. Whereas, when the GRK2 Bacman® was transduced into cells a 150-fold increase in the level of GRK2 mRNA was observed.

## Discussion

In this study we have revealed that co-expression of GRK2 in a µ-opioid U2OS β-arrestin cell line, renders the receptor in a state that is pharmacologically distinct from that of the receptor in the absence of GRK2 co-expression. Understanding the interaction of receptors with both ligands and effector molecules is an important process in drug discovery. There are a number of models which describe this interaction. An important differentiator between models is whether they describe the receptor as an allosteric protein i.e. can its conformation be changed by the interaction of another protein (signalling molecule). When determining agonist bias changes in the affinity, as well as the efficacy of the ligand, must be taken into consideration [25]. A non-allosteric model describes a single affinity of an agonist for the receptor and attributes bias purely to differences in efficacy. However, an allosteric model assumes that the interaction of a signalling molecule with the receptor affects agonist affinity and therefore a biased compound may exhibit changes in affinity and efficacy. This study supports the hypothesis that the affinity of agonists is directly related to the effector-specific conformation of the receptor, which in turn is a result of the allosteric nature of GPCRs and the cellular environment in which they exist.

Co-expression of GRK2 in U20S µ-opioid β-arrestin cells led to an increase in the intrinsic activity of very weak partial agonists and a >10 fold increase in the potency of all agonists (whether full or partial initially). The linear relationship between occupancy and response in the β-arrestin assay, exemplified by the lack of effect of decreasing receptor number on compound potency, means that the effect of GRK2 co-expression is reflecting a change in agonist affinity, rather than system amplification. Under conditions in which there was no GRK2 co-expression there was good correlation between K_L_ in binding and the β-arrestin EC_50_ (Nickolls et al., 2011 and data herein). However, although we saw a small increase in agonist affinity in membrane preparations from co-expressing cells under conditions designed to measure K_L_, this was not as great as the change in agonist EC_50_ in the β-arrestin assay. This could be due to the lack of β-arrestin/receptor complex in the membrane preparation compared to the situation in whole cells (in which β-arrestin is recruited upon agonist binding). There was a slight increase in basal β-arrestin recruitment in the cells co-expressing GRK2, which could explain the slight change in binding affinity. However, it is considered that once membranes are prepared, the system is fixed and β-arrestin cannot be recruited. As such the receptor conformation detected in the functional assay cannot be measured in the binding assay. This hypothesis is consistent with the work of Mary et al., [26], who have shown that β-arrestin-2 stabilizes a ghrelin receptor conformation which is significantly different to that stabilized by agonist binding, and in addition this conformation of the receptor is not detectable in the absence of β-arrestin-2. Additionally, Krasel and colleagues [27] reported that upon agonist removal, β-arrestin dissociated rapidly from phosphorylated β-adrenergic receptors, making it unlikely that the complex would exist in membranes prepared from cells in the absence of agonist.

The allosteric effect of receptor molecules on receptor conformation is an important consideration when determining efficacy. Substitution of a binding K_i_ value into the operational model of agonism [22] to determine tau, is therefore not supported by our data, as affinity is dependent on the effector molecule present. Additional data supporting effector-specific receptor conformations is emerging. SCAM analysis has revealed that the κ-opioid receptor changes conformation differentially in TM 6 and 7, dependent on whether Gαi2 or Gα16 is co-expressed [28]. Furthermore, different conformational states of the CCK2 receptor have been revealed dependent on whether the effector molecule is β-arrestin or Gq [29]. In our previous study, we used receptor depletion coupled with the operational model of agonism to allow both tau and K_A_ to be determined by functional data [23] and consistent with the conclusions of this study, functionally determined K_A_ values were not equivalent to Ki values determined in binding.

In comparison to brain frontal lobe GRK levels, the amount of overall GRK expression was very low in µ-opioid β-arrestin U2OS cells, where the only GRK detected at a reasonable level was GRK6. If all GRKs are able to increase agonist potency in a similar fashion to GRK2, it is likely that the U2OS µ-opioid β-arrestin cell line under-predicts agonist-induced β-arrestin recruitment compared to the in vivo situation simply due to the low expression of GRKs. The difference in agonist potency caused by the co-expression of GRK2 indicates that in the absence of GRK2 the receptor state interacting with β-arrestin is a different receptor conformation, this could be caused by either the receptor-GRK2 allosteric complex or GRK2 mediated phosphorylation of different receptor residues. Consistent with this it has been reported that different GRKs produce distinct phosphorylation patterns of the µ-opioid receptor. GRK2 has been reported to phosphorylate the µ-opioid receptor on both threonine 370 and serine 375 [10], [11], whereas GRK5 only phosphorylates on serine 375 [10].

The effect of pertussis toxin on β-arrestin recruitment also varied depending on whether or not GRK2 was co-expressed. In the absence of GRK2 co-expression β-arrestin recruitment was partially PTX sensitive, indicating that some of this recruitment was G protein dependent. In the presence of GRK2 there was no PTX sensitivity. It is reported that Gβγ is usually required to recruit/localise GRK2 at the plasma membrane [30], [31], which explains the PTX sensitivity of the β-arrestin recruitment in the absence of GRK2 expression. We consider that our data show that when GRK2 is highly over-expressed, a proportion of GRK2 is already localised at the plasma membrane and the need for Gβγ recruitment is superseded.

The effect of GRK2 co-expression on agonist Emax in the β-arrestin assay was not consistent. Although the compounds which showed weak intrinsic activity in the absence of GRK2 (morphine and oxycodone) showed full agonist properties when GRK2 was co-expressed. The higher intrinsic activity partial agonists (endomorphin-1 and -2), did not show any change in their Emax. We consider it interesting that endomorphin-2 differs from the majority of other compounds, as although we disagree with the methodology the authors used to calculate bias, it has been classified as a β-arrestin biased compound [32]. Consequently, it would be interesting to see what effect co-expression of GRK2 had on the G protein biased compound TRV130 [19]. As TRV130 shows some recruitment of β-arrestin in the absence of GRK2 co-expression, all be to a much lesser degree than morphine, we would theorise that it may recruit more β-arrestin in the presence of GRK2. This would be consistent with the fact that although its therapeutic index is much improved with respect to morphine, it still does produce constipation and respiratory depression in preclinical species.

In both the β-arrestin assay and the [^35^S]GTPγS binding functional assay a decrease in maximal assay window was observed. This could be partially accounted for by the cytotoxic effect of Bacman® transduction, as shown by the control GFP transduction experiments. However, it is considered that it is likely that GRK2 itself is sterically impeding second messenger recruitment. Consistent with this hypothesis, the ability of morphine to inhibit adenylate cyclase was attenuated by co-expression of GRK2 [15]. Furthermore, β-arrestin has been shown to compete with GRK2 for interaction with the glucagon-like peptide-1 receptor [33].

The inverse agonist properties of naloxone were clearly visible in the [^35^S]GTPγS binding assay when GRK2 was co-expressed. Naloxone has been shown to exhibit inverse agonist properties in vitro [34] and in vivo after chronic agonist treatment [35] and it is hypothesised that chronic agonist treatment leads to agonist independent constitutive activity of the µ-opioid receptor. Furthermore, Birdsong and colleagues [36] have observed an increase in agonist affinity for the µ-opioid receptor after prolonged agonist exposure, suggestive of the receptors exhibiting memory.

Recently it has been proposed that different GRKs cause varied phosphorylation patterns on receptors [37], which can act as a “barcode” in determining which specific cellular signalling pathways are activated. We are therefore currently continuing our work by investigating the effect of GRK5 (which falls into the GRK4 like family and like GRK2 is ubiquitously expressed) on µ-opioid agonist affinity and efficacy. Additionally we are interested as to whether similar effects are observed at the other opioid receptors. The current data are limited by the fact that we are studying ligands in a recombinant system, but we hope that the dataset we build across a diverse chemical space will allow us to discover tools, which can be used in vivo, to assess the translatability of our system and the effects of agonist bias in vivo.

Overall our study is consistent with ligands and effector molecules stabilizing unique conformations of the allosteric µ-opioid receptor. This has implications in drug discovery when describing how ligands act at receptors and in determining efficacy.
